# “*From partnered steps to purposeful engagement*” higher-order, dimension-level, and importance-performance map analyses of the tripartite model of individual interest and study engagement in ballroom-based physical education

**DOI:** 10.3389/fspor.2026.1807816

**Published:** 2026-09-09

**Authors:** Joseph Lobo

**Affiliations:** College of Sports, Exercise and Recreation, Bulacan State University, Malolos, Philippines

**Keywords:** ballroom dancing, individual interest, motivation, physical education, study engagement

## Abstract

**Background:**

Ballroom dance remains a contested domain in Philippine higher education, often perceived as formal, outdated, or misaligned with the values and movement preferences of contemporary learners. Within physical education, this creates a need to examine how students' individual interest relates to their study engagement in ballroom-based learning contexts.

**Objective:**

This cross-sectional study examined the association between individual interest and study engagement in ballroom-based physical education, using the tripartite model of individual interest comprising positive affect and willingness to reengage, stored utility value, and stored attainment value and knowledge-seeking intentions.

**Methods:**

A total of 138 Filipino college students enrolled in PATH-FIT 4 participated in the study. Data were analyzed using partial least squares structural equation modeling (PLS-SEM). Individual interest was first modeled as a reflective-reflective higher-order construct, followed by a dimension-level model examining the separate associations of the three individual interest dimensions with study engagement. An importance-performance map analysis (IPMA) was also conducted to identify the practical relevance of each dimension in relation to study engagement.

**Results:**

The higher-order model showed that individual interest was positively associated with study engagement and accounted for 49.3% of its variance. In the dimension-level model, stored utility value showed the strongest positive association with study engagement, followed by stored attainment value and knowledge-seeking intentions. Positive affect and willingness to reengage was not significantly associated with study engagement after accounting for the other dimensions.

**Conclusion:**

The findings suggest that engagement in ballroom-based PE is linked less to affective enjoyment alone and more to students' recognition of ballroom dance as useful, personally meaningful, and connected to further learning. Positioning ballroom pedagogy around usefulness, self-relevance, cultural meaning, and knowledge-seeking may help PE instructors present dance-based learning as a more purposeful movement experience in Philippine HE.

## Introduction

Engaging students in movement-based courses within higher education has become increasingly complex, especially as many tertiary learners now perceive physical education (PE) as peripheral to academic success rather than central to holistic development (1–3). This concern is visible across international tertiary PE contexts and remains relevant in the Philippines, where issues of implementation, student receptivity, and curricular relevance continue to shape the status of PE in higher education (4, 5). Dance-based PE appears particularly vulnerable to these challenges because students may enter dance classes with unfamiliarity, discomfort, disinterest, or resistance (6–9). Ballroom dance occupies a more complicated position within this landscape. Although it can support rhythm, posture, coordination, non-verbal communication, discipline, social interaction, and embodied learning (10, 11), it is often perceived by contemporary students as formal, outdated, culturally distant, or misaligned with youth-oriented movement preferences. This creates a gap between the curricular value attributed to ballroom dance and the ways students actually receive, interpret, and engage with it.

### Ballroom-based physical education and engagement tensions in higher education

The challenge of ballroom-based PE is not simply that students are uninterested in movement or dance. Rather, disengagement may emerge from the way ballroom is framed, taught, assessed, and positioned within higher education. Unlike many individual PE activities, ballroom requires students to coordinate movement with a partner, follow rhythmic and spatial patterns, perform socially readable gestures, and participate in tasks where bodily confidence, peer interaction, and public performance become difficult to separate from learning. These features make ballroom a socially exposed movement context, as students are not only learning steps but also managing proximity, timing, coordination, correction, and the possibility of being watched by others. Ballroom may also carry gendered and culturally familiar movement expectations, particularly when instruction relies on fixed partner roles or formal bodily presentation (12–15). For this reason, ballroom-based PE offers a useful setting for examining how different dimensions of individual interest are associated with study engagement.

In several higher education contexts, including the Philippines, ballroom-based PE may also reflect broader tensions between traditional curricular expectations and contemporary student preferences (16). Although ballroom holds physical, social, artistic, and cultural value, students may perceive it as formal, unfamiliar, outdated, or less aligned with the expressive, informal, digital, and personalized movement cultures they encounter outside school (17, 18). This tension becomes pedagogically important when ballroom is presented mainly as technical reproduction or performance compliance rather than as a meaningful movement-learning experience. Scholarship on dance in PE similarly suggests that students' discomfort often comes not from dance itself, but from how dance is organized, evaluated, and embodied in class (6–9). Within this study, these contextual features are not treated as separate explanatory variables. Rather, they provide the background for examining whether students' affective enjoyment, perceived usefulness, and self-relevant knowledge-seeking are associated with their engagement in ballroom-based PE.

### Individual interest as a motivational lens in physical education

Individual interest offers a useful theoretical lens for examining this issue because it explains how students develop relatively enduring connections to learning content beyond momentary enjoyment. Roure et al. (19) conceptualized individual interest in PE as a tripartite construct composed of positive affect and willingness to reengage (PAWR), stored utility value (SUV), and stored attainment value and knowledge-seeking intentions (SAVKSI). These dimensions capture affective, instrumental, and self-relevant forms of motivation, thereby extending interest beyond simple preference, liking, or enjoyment (20). This framework is especially relevant for ballroom-based PE because students may experience the activity in multiple ways at once. Some may enjoy the movement, others may value its health or social benefits, while others may connect it to confidence, cultural knowledge, self-expression, or personal development.

The tripartite model of individual interest serves as the primary conceptual framework of the present study because it directly organizes the three motivational factors examined in the model. The other theories are used as supporting lenses rather than as additional tested frameworks. Expectancy-Value Theory helps clarify why SUV and SAVKSI may be relevant to engagement, as students are more likely to invest effort in learning activities, they perceive as useful, important, or connected to their goals (21). Self-Determination Theory supports the interpretation that students' engagement may be strengthened when ballroom-based PE allows them to experience competence, autonomy, and relatedness within the learning environment (22, 23). Situated Learning Theory further explains why these motivational processes should be understood in relation to the specific social and cultural organization of ballroom instruction, including partner interaction, bodily visibility, rhythm, proximity, and classroom participation norms (24). In this way, the tripartite model provides the structure of the empirical analysis, while EVT, SDT, and Situated Learning Theory help explain why the three dimensions of individual interest may be associated with study engagement in ballroom-based PE (25–27).

### Study engagement in movement-based learning

Study engagement provides the outcome lens for understanding how individual interest relates to students' learning investment in ballroom-based PE. Drawing from Carmona-Halty et al. (28), this refers to a positive and study-related state characterized by vigor, dedication, and absorption, extending the broader work engagement tradition into academic settings (29, 30). In PE, however, engagement cannot be reduced to attendance, compliance, or task completion. Movement-based learning involves bodily exposure, social interaction, physical effort, affective response, and skill development, making engagement highly sensitive to task meaning, peer dynamics, perceived competence, autonomy support, and instructional design (31–33).

This sensitivity becomes more pronounced in dance-based PE because students are asked to learn through bodies that are visible, evaluated, culturally interpreted, and socially positioned. In ballroom dance, engagement may be shaped by enjoyment, embarrassment, perceived usefulness, curiosity, partner interaction, social comparison, bodily confidence, and gendered expectations. For this reason, individual interest and study engagement should be examined together, not simply to determine whether students like ballroom, but to understand what forms of interest are associated with deeper investment in the learning experience. When ballroom is perceived as useful, personally meaningful, and connected to further learning, students may be more likely to engage with it as an educational experience rather than as a mere performance requirement.

### Empirical gap and contribution of the present study

Despite growing attention to motivation and engagement in PE, ballroom-based PE remains underexamined as a context for applying the tripartite model of individual interest. International scholarship on interest and engagement suggests that students' investment in learning is shaped not only by enjoyment, but also by perceived usefulness, personal relevance, autonomy support, teacher behavior, peer interaction, bodily confidence, and the way learning tasks are framed as meaningful (31, 34–39). In PE, earlier work has similarly shown that interest is connected to task design, perceived value, learning goals, and students' broader movement experiences (22, 40–42). Within this broader literature, individual interest provides a focused motivational lens because it captures not only affective enjoyment, but also perceived usefulness, personal relevance, and knowledge-seeking orientation (19, 20, 43, 44). Scholarly studies in the Philippines have begun to validate and apply this framework across selected PE contexts, including general PE, recreational games, dance-based activities, individual and dual sports, martial movement, and gymnastics-based learning (45–54). However, these studies also suggest that the dimensions of individual interest may not relate to engagement in the same way across movement contexts. In some activities, affective enjoyment may be more visible, while in others, perceived usefulness, attainment value, or knowledge-seeking may carry stronger engagement-related relevance. Therefore, ballroom-based PE remains a meaningful gap because it combines partnered movement, social exposure, formal bodily presentation, rhythm, gendered conventions, and cultural meanings in ways that may shape how the three factors of individual interest are associated with study engagement.

Addressing this gap is theoretically and pedagogically important. Theoretically, the study applies Roure et al.'s (19) tripartite model to a socially and culturally distinct dance context within Philippine HE. Ballroom-based PE provides a useful context for examining whether the three factors of individual interest operate similarly or differently when the learning activity involves partnered movement, bodily visibility, rhythm, proximity, and performance-related exposure. By analyzing individual interest both as a higher-order construct and through its separate dimensions, the study moves beyond asking whether students are generally interested in ballroom dance. It instead examines which aspects of interest are most closely associated with study engagement. This is important because the three factors of individual interest may carry different motivational weight depending on how a PE activity is experienced, valued, and socially organized.

Pedagogically, the study provides evidence that may help PE instructors reframe ballroom beyond nostalgia, technical compliance, or aesthetic performance, and toward a more meaningful, value-centered, and learner-responsive movement experience. Such evidence is needed because instructors may otherwise rely on broad assumptions that students disengage from ballroom because it is old, difficult, or irrelevant, rather than examining which dimensions of interest are actually associated with engagement. If engagement is more strongly linked to perceived usefulness, self-relevance, and knowledge-seeking than to affective enjoyment alone, then ballroom pedagogy may need to foreground why the activity matters for students' bodies, confidence, social interaction, cultural understanding, and continued movement learning. At the same time, these pedagogical implications should be understood as interpretive applications of the findings rather than direct causal conclusions, given the cross-sectional design of the study.

### Objectives and hypotheses formulation

This cross-sectional study examined the association between students' individual interest in ballroom dance and their study engagement in ballroom-based PE. Specifically, individual interest was analyzed in two complementary ways. First, it was modeled as a reflective-reflective higher-order construct (HOC) represented by PAWR, SUV, and SAVKSI. Second, the separate dimension-level associations of the three factors of individual interest with study engagement were examined to identify which components of individual interest were most strongly linked to engagement. Guided by Roure et al. (19) tripartite model, the study hypothesized that overall individual interest would be positively associated with study engagement. It was also hypothesized that PAWR, SUV, and SAVKSI would each show positive associations with study engagement. By examining these associations, the study aims to clarify whether ballroom-based PE engagement is more closely linked to affective enjoyment, perceived usefulness, self-relevance, knowledge-seeking, or the broader configuration of individual interest as a multidimensional motivational construct.

## Methods

### Participants

The participants of this study were 138 s-year undergraduate students enrolled in PATH-FIT 4, or Physical Activity Towards Health and Fitness, during the second semester of Academic Year 2024–2025 at a public university in the Philippines. PATH-FIT 4 is a required course under the university's implementation of the revised General Education curriculum and the Revitalized Tertiary Physical Education Program. In the participating class section, ballroom dance served as the primary instructional content, reflecting the specialization of the assigned Physical Education instructor. This context provided a structured, partner-based movement experience that was directly aligned with the focus of the study. The participants came from different academic programs, allowing the study to capture responses from students with varied disciplinary backgrounds while maintaining a common exposure to ballroom dance instruction.

To determine the minimum required sample size, an *a priori* power analysis was conducted using G*Power version 3.1 (55). Because the dimension-level structural model included three lower-order dimensions of individual interest associated with study engagement, the calculation was based on three exogenous constructs directed toward the endogenous construct. Parameters were set to detect a medium effect size (*f*^2^ = .15) at a .05 significance level with .95 statistical power. The analysis indicated a minimum required sample of 119 participants. With 138 students included in the final sample, the study exceeded the minimum requirement for the planned PLS-SEM structural analysis. Although the achieved sample exceeded the minimum required sample from the *a priori power analysis*, it remains modest in relation to the HOC specification, dimension-level model, and IPMA conducted in this study. For this reason, the estimates were interpreted with caution and were evaluated alongside measurement quality indicators, collinearity diagnostics, bootstrapped confidence intervals, and effect size estimates. The higher-order and dimension-level models were treated as complementary analyses for clarifying the structure and engagement-related associations of individual interest rather than as definitive evidence of model stability across broader populations. The demographic profile of the participants is presented in Table 1. In terms of sex/gender identity, 53 students identified as heterosexual men (38.4%), 74 as heterosexual women (53.6%), 9 as LGBTQIAP+ (6.5%), and 2 preferred not to disclose (1.4%). Participants’ ages ranged from 19 to 28 years, with a mean ($\bar{x}$) age of 21.07. The majority were 19 years old (45.7%) or 20 years old (18.1%).

**Table 1:** Profile distribution of the participants (*n* = 138).

Demographic profile	Items	*n*(%)
Sex/gender identity	Heterosexual men	53 (38.4)
	Heterosexual women	74 (53.6%)
	LGBTQIAP+	9 (6.5)
	Prefer not to disclose	2 (1.4)
Age ($\bar{x}=21.07$)^a^	19	63 (45.7)
	20	25 (18.1)
	21	7 (5.1)
	22	3 (2.2)
	23	12 (8.7)
	24	9 (6.5)
	25	4 (2.9)
	26	6 (4.3)
	27	3 (2.2)
	28	6 (4.3)

a Mean age.

### Instruments

Data were collected through an online survey administered via Google Forms throughout May 2025, after the completion of the semester and the students' formal exposure to ballroom dance instruction in PATH-FIT 4. The questionnaire consisted of three sections. The first section gathered demographic information, including age and sex/gender identity. The second section used the Students' Individual Interest in Physical Education Questionnaire adapted from Roure et al. (19). The original instrument measures individual interest through three theoretically supported dimensions: PAWR, SUV, and SAVKSI. Items were rated using a 5-point Likert scale ranging from 1- “strongly disagree” to 5- “strongly agree.” Minor contextual wording adaptations were made to align the items with the ballroom-based PE context, such as changing references to “physical education” into “ballroom class.” These adaptations were treated as content-specific contextual modifications rather than a redevelopment of the instrument. The wording changes were limited to replacing the general PE referent with the specific ballroom-based PE context while retaining the original conceptual meaning of each dimension. Thus, items measuring PAWR continued to refer to students' positive affective responses and willingness to participate again, items measuring SUV continued to refer to perceived usefulness, and items measuring SAVKSI continued to refer to personal importance and interest in learning more. The use of the instrument was also informed by prior Philippine validation work on the tripartite model of individual interest in PE, which supported the relevance of the its three factors for examining Filipino students' motivational connection to PE learning contexts (52). Because the present study applied the instrument to a specific ballroom-based PE course, the adequacy of the adapted items was further examined through the subsequent PLS-SEM measurement model assessment. The third section included the Utrecht Work Engagement Scale for Students-Short Form (UWES-9S) developed by Carmona-Halty et al. (28). The UWES-9S assesses study engagement through vigor, dedication, and absorption. Items were rated using a 7-point frequency scale ranging from 0- “never” to 6- “always.” In the present study, study engagement was treated as an overall latent construct, consistent with the use of UWES as a global indicator of students' energy, dedication, and absorption in academic learning.

#### Instrument reliability, validity, and contextual application

The reliability and validity of the adapted measurement model were assessed to determine whether the contextually modified items retained acceptable psychometric quality in the ballroom-based PE setting. All constructs were specified as reflective constructs in accordance with the theoretical structure of the instruments. Indicator reliability was examined through outer loadings, with indicators below the recommended threshold of ≥.70 removed from the final model to improve measurement quality. Accordingly, Table 2 presents only the retained indicators included in the final measurement model. Internal consistency reliability was evaluated using Cronbach's alpha (α) and composite reliability (CR), while convergent validity was assessed using the average variance extracted (AVE). Following Hair et al. (56), α and CR values ≥.70 and AVE values ≥.50 were considered acceptable. As shown in Table 2, all retained indicators demonstrated adequate outer loadings, ranging from .716 to .942. The constructs also showed satisfactory internal consistency, with α values ranging from .889 to .935 and CR values ranging from .894 to .935. Convergent validity was likewise supported, with AVE values ranging from .649 to .884, exceeding the recommended minimum threshold of ≥.50. Indicator-level variance inflation factor (VIF) values were below the conservative threshold of <5.0, indicating no critical collinearity concerns among the retained indicators. These results support the internal consistency reliability, indicator reliability, and convergent validity of the adapted measures for examining individual interest and study engagement in ballroom-based PE.

**Table 2:** Measurement model assessment and convergent validity metrics.

Construct	Items	Item loadings	CA	CR	AVE	VIF (Outer model)
PAWR	PAWR1	0.867	0.903	0.905	0.720	2.827
	PAWR2	0.835				2.446
	PAWR3	0.896				3.238
	PAWR4	0.821				2.055
	PAWR5	0.823				2.042
SUV	SUV1	0.851	0.889	0.894	0.751	2.233
	SUV2	0.835				2.070
	SUV3	0.890				2.655
	SUV4	0.890				2.689
SAVKSI	SAVKSI1	0.938	0.935	0.935	0.884	3.877
	SAVKSI2	0.941				3.986
	SAVKSI3	0.942				4.058
UWES	UWES1	0.716	0.909	0.914	0.649	1.994
	UWES3	0.850				3.033
	UWES4	0.806				2.467
	UWES5	0.791				2.256
	UWES6	0.834				2.587
	UWES7	0.862				3.221
	UWES9	0.769				2.044

Item loadings >0.70, Cronbach's Alpha (CA) and Composite Reliability (CR) >0.70, AVE (Average Variance Extracted) >0.50, VIF (Variance Inflation Factor) <5.0.

PAWR, Positive affect and willingness to reengage; SUV, Stored-utility value; SAVKSI, Stored attainment value and knowledge-seeking intentions; UWES, Study engagement.

Discriminant validity was assessed using the Fornell-Larcker criterion and the Heterotrait-Monotrait Ratio (HTMT), following the recommendations of Hair et al. (56). As presented in Table 3, the square roots of the AVE values for each latent construct, shown on the diagonal, exceeded their corresponding inter-construct correlations. This satisfied the Fornell-Larcker criterion and indicated that each construct shared more variance with its own indicators than with other constructs in the model (57). In addition, the HTMT values ranged from .635 to .813, remaining below the conservative threshold of <.85 recommended by Henseler et al. (58). These results support the discriminant validity of the measurement model and suggest that the contextual wording adaptations did not compromise the empirical distinctiveness of PAWR, SUV, SAVKSI, and UWES in the present sample.

**Table 3:** Fornell–larcker and HTMT discriminant validity assessment for latent constructs.

Fornell-larcker criterion				
	PAWR	SAVKSI	SUV	UWES
PAWR	0.849			
SAVKSI	0.714	0.940		
SUV	0.730	0.699	0.867	
UWES	0.581	0.640	0.692	0.805

Heterotrait-monotrait ratio (HTMT)				
PAWR				
SAVKSI	0.776			
SUV	0.813	0.770		
UWES	0.635	0.690	0.763	

Diagonal values (bold) indicate the square root of AVE; values below the diagonal reflect inter-construct correlations. HTMT ratio value <0.85 (conservative) <0.90 (liberal) approach.

PAWR, Positive affect and willingness to reengage; SUV, Stored-utility value; SAVKSI, Stored attainment value and knowledge-seeking intentions; UWES, Study engagement.

#### Common method bias assessment

Common method bias was assessed because all constructs were measured using a single online self-report questionnaire administered at one point in time. As a collinearity-based diagnostic, the inner model VIF values in the dimension-level model were examined. The VIF values for PAWR, SUV, and SAVKSI were 2.593, 2.482, and 2.367, respectively. These values were below the conservative 3.3 threshold commonly used as a diagnostic indicator for potential common method bias in PLS-SEM. This suggests that common method bias was unlikely to be a serious concern in the dimension-level model. Nevertheless, because the study relied on cross-sectional self-report data, method-related bias cannot be fully ruled out.

### Data analysis

Data analysis was conducted in two phases. First, demographic data were encoded and summarized using IBM SPSS Statistics. Frequencies and percentages were computed for categorical demographic variables, including sex/gender identity, while age was summarized using frequency distribution and mean value. These descriptive results were used only to present the demographic profile of the participants. Second, partial least squares-structural equation modeling (PLS-SEM) was conducted using SmartPLS 4 to assess the measurement and structural models. PLS-SEM was selected because the study focused on latent construct associations, explained variance, dimension-level contributions, and the practical relevance of the individual interest dimensions without making causal claims. This approach was also appropriate because the analysis included a reflective-reflective HOC, a dimension-level structural model, and importance-performance map analysis (IPMA). Although covariance-based SEM is commonly used for theory confirmation and global model fit assessment, PLS-SEM was more aligned with the present study's focus on component-based modeling, construct reliability and validity, bootstrapped path estimates, effect sizes, and explained-variance estimation in a modest sample. Prior to structural model testing, the reflective measurement model was evaluated through indicator reliability, internal consistency reliability, convergent validity, and discriminant validity.

To align the analysis with Roure et al.'s (19) tripartite model, two complementary structural models were tested. The first model specified individual interest as a reflective-reflective HOC represented by PAWR, SUV, and SAVKSI, with UWES as the endogenous construct. The second model examined the dimension-level associations of PAWR, SUV, and SAVKSI with UWES to determine which components of individual interest were most strongly associated with study engagement in ballroom-based PE. Path significance was evaluated using bootstrapping with 10,000 subsamples. Structural model results were interpreted using standardized path coefficients, standard errors, *t*-values, *p*-values, confidence intervals (CIs), coefficients of determination, and *f*^2^ effect sizes. The coefficient of determination was used to assess the explained variance in study engagement, while *f*^2^ was used to determine the relative contribution of each exogenous construct. IPMA was then conducted for the dimension-level model, with study engagement specified as the target construct. Importance values were based on total effects, while performance values were interpreted using rescaled construct scores based on the theoretical response range of the individual interest scale.

### Ethical statement

This study was conducted in accordance with ethical standards for research involving human participants. Prior to data collection, ethical clearance was sought from the Local Research Ethics Committee of the College of Sports, Exercise and Recreation. The study was approved due to its minimal risk nature, reliance on anonymous self-report questionnaires, and non-invasive procedures. Participation was voluntary, informed consent was obtained digitally from all respondents, and no personally identifiable information was collected.

## Results

The reflective-reflective HOC evaluation supported the specification of INDINT as a multidimensional construct. As shown in Table 4, all lower-order constructs loaded strongly and significantly on the HOC of INDINT. PAWR showed the highest loading [λ = .920, SE = .015, *t* = 60.080, *p* < .001, 95% CI (.887, .947)], followed by SUV [λ = .899, SE = .022, *t* = 40.385, *p* < .001, 95% CI (.850, .936)] and SAVKSI [λ = .879, SE = .022, *t* = 39.976, *p* < .001, 95% CI (.831, .917)]. These findings indicate that the affective-reengagement, utility-value, and attainment-value and knowledge-seeking dimensions functioned as strong reflective dimensions of INDINT in ballroom-based PE.

**Table 4:** Higher-order construct evaluation of individual interest.

HOC	LOC	Loading λ	SE	*t*	*p*	95% CI [LL, UL]
INDINT	PAWR	0.920	0.015	60.080	<.001	[0.887, 0.947]
	SUV	0.899	0.022	40.385	<.001	[0.850, 0.936]
	SAVKSI	0.879	0.022	39.976	<.001	[0.831, 0.917]

The values represent standardized reflective loadings of the lower-order constructs on the higher-order construct. All loadings were statistically significant at *p* < .001.

HOC, higher-order construct; LOC, lower-order construct; SE, standard error; CI, confidence interval; LL, lower limit; UL, upper limit. INDINT, individual interest; PAWR, positive affect and willingness to reengage; SUV, stored utility value; SAVKSI, stored attainment value and knowledge-seeking intention.

### Structural model assessment

**Figure 1:**
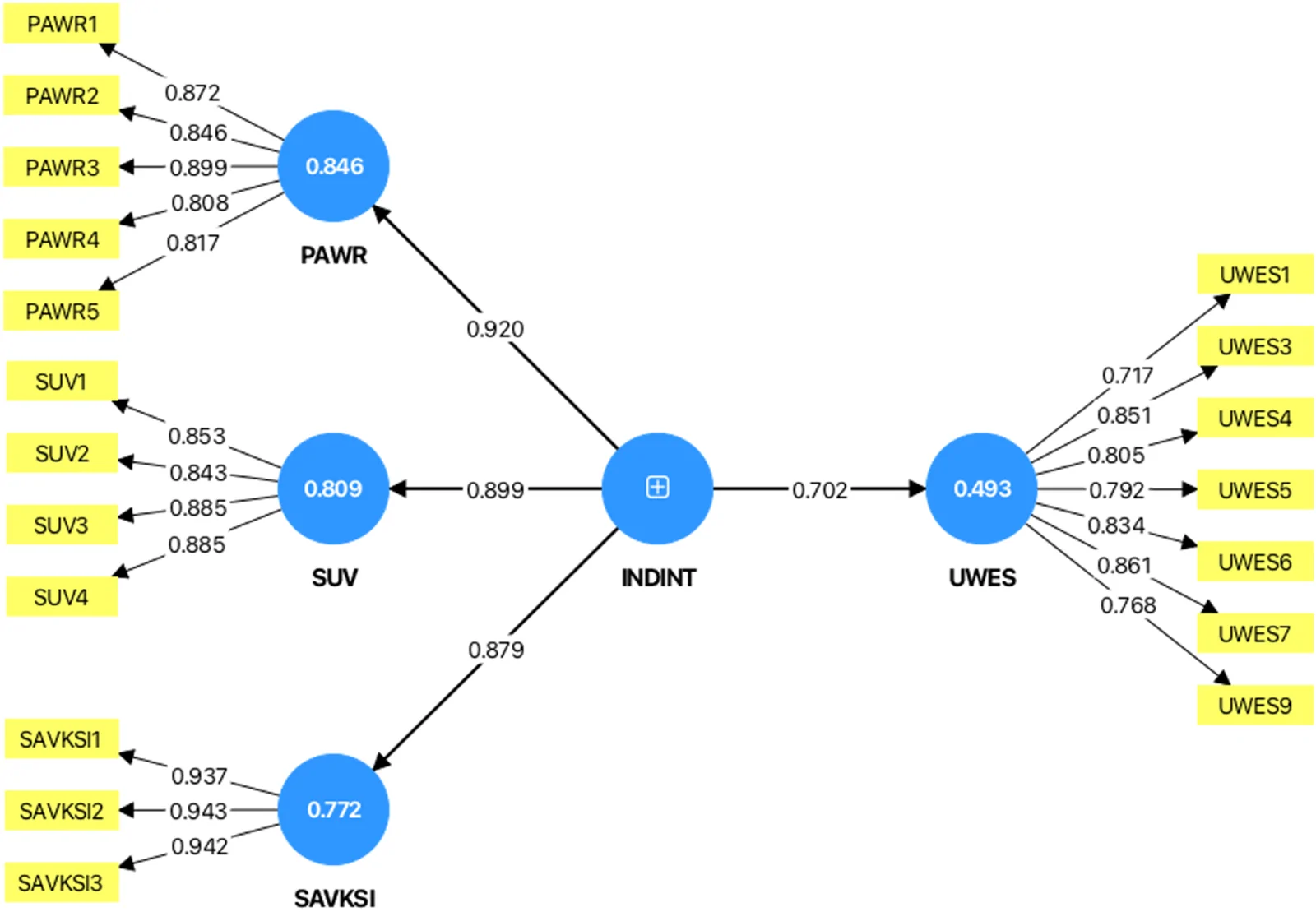
Higher-order structural model of individual interest and study engagement in ballroom-based PE.

The explanatory power of the higher-order structural model was assessed using the coefficient of determination (R^2^) of the endogenous construct. As shown in Figure 1, the model explained 49.3% of the variance in study engagement (R^2^ = .493), indicating moderate explanatory power.

The higher-order structural model was then evaluated by examining the path between INDINT and UWES. As presented in Table 5, INDINT showed a positive and statistically significant association with UWES [*β* = .702, SE = .047, *t* = 15.063, *p* < .001, 95% CI (.608, .790)]. The confidence interval did not include zero. The effect size was large (*f*^2^ = .97). Because the higher-order model included only one exogenous construct, the *f*^2^ value represents the total explanatory contribution of INDINT in this model and should be interpreted alongside the R^2^ value.

**Table 5:** Structural model results.

Path	*β*	SE	*t-*value	*p*-value	R^2^	*f^2^*	95% CI [LL, UL]	Decision
INDINT → UWES	0.702	0.047	15.063	<.001	0.493	0.97	[0.608, 0.790]	Supported

Path significance was determined using bootstrapping with 10,000 subsamples. Because the higher-order model included only one exogenous construct, the *f^2^* value represents the total explanatory contribution of individual interest to study engagement and should be interpreted alongside the R^2^ value.

*β,* standardized path coefficient; SE, standard error; R^2^, coefficient of determination; *f^2^*, effect size; CI, confidence interval; LL, lower limit; UL, upper limit.

### Additional analysis of dimensional contributions to study engagement

**Figure 2:**
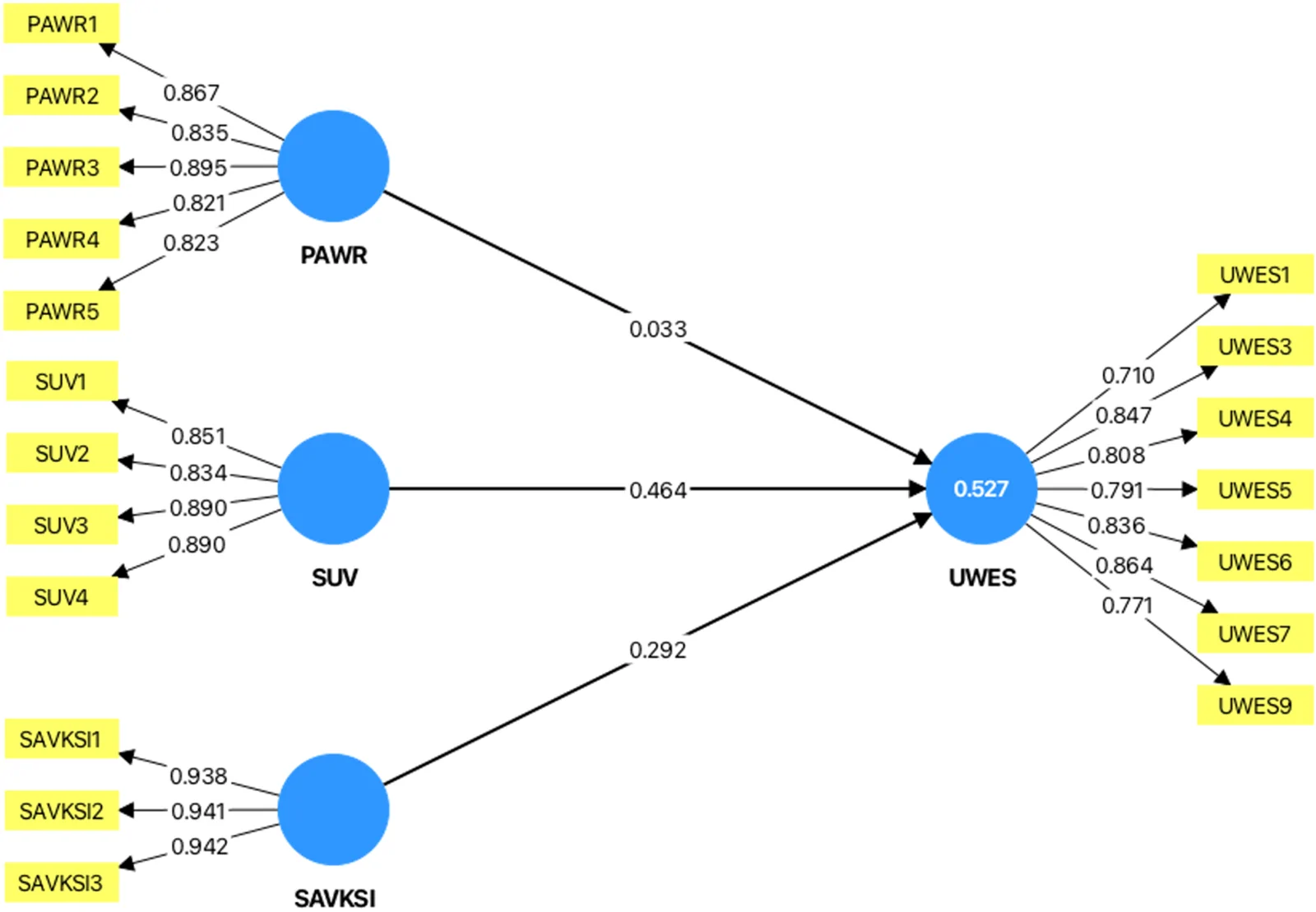
Dimension-level model of individual interest and study engagement in ballroom-based PE.

Before interpreting the dimension-level structural paths, inner model collinearity was examined to determine whether PAWR, SUV, and SAVKSI could be entered simultaneously in the model. As reported in the CMB assessment, all VIF values were below the conservative <3.3 threshold. These results indicate that no critical collinearity concerns were present among the three lower-order dimensions of INDINT and that they were not statistically redundant as simultaneous exogenous constructs in the dimension-level model. To further examine the dimension-specific contributions of INDINT, an additional structural model was estimated in which PAWR, SUV, and SAVKSI were directly associated with study engagement. As shown in Figure 2, the model explained 54.2% of the variance in UWES (R^2^ = .542), indicating that the three lower-order dimensions of INDINT accounted for a meaningful proportion of students' reported engagement in ballroom-based PE. As presented in Table 6, SUV showed the strongest positive association with study engagement [*β* = .464, SE = .094, *t* = 4.950, *p* < .001, 95% CI (.298, .666)], followed by SAVKSI [*β* = .292, SE = .105, *t* = 2.788, *p* = .005, 95% CI (.071, .482)]. Both CI did not include zero, supporting the stability of the estimated associations. These findings indicate that students who perceived ballroom dance as useful, personally meaningful, and connected to further learning tended to report higher UWES. In contrast, PAWR was not significantly associated with study engagement [*β* = .033, SE = .102, *t* = .319, *p* = .750, 95% CI (−.164, .234)]. The CI included zero, indicating that PAWR did not show an independent association with UWES after accounting for SUV and SAVKSI. Thus, the dimension-level model suggests that the relationship between INDINT and UWES in ballroom-based PE was more strongly linked to students’ perceived utility, attainment value, and knowledge-seeking orientation than to affective enjoyment alone.

**Table 6:** Dimension-level structural model results.

Path	*β*	SE	*t-*value	*p*-value	*f^2^*	95% CI [LL, UL]	Decision
PAWR → UWES	0.033	0.102	0.319	0.750	0.00	[−0.164, 0.234]	Not supported
SUV → UWES	0.464	0.094	4.950	<.001	0.18	[0.298, 0.666]	Supported
SAVKSI → UWES	0.292	0.105	2.788	.005	0.08	[0.071, 0.482]	Supported

Path significance was determined using bootstrapping with 10,000 subsamples, while *f^2^* values were obtained from the PLS Algorithm results.

*β,* standardized path coefficient; SE, standard error; *f^2^*, effect size; CI, confidence interval; LL, lower limit; UL, upper limit.

PAWR, positive affect and willingness to reengage; SUV, stored utility value; SAVKSI, stored attainment value and knowledge-seeking intention; UWES, study engagement.

### Importance-performance map analysis results

IPMA was conducted using UWES as the target construct to examine the importance and performance values of the three INDINT dimensions in the dimension-level model. As shown in Table 7, SUV had the highest importance value (.464) and a performance score of 71.75. SAVKSI had an importance value of.292 and the highest performance score of 81.00. PAWR had the lowest importance value (.033) and a performance score of 70.50.

**Table 7:** Importance-performance map analysis (IPMA) results for study engagement.

Construct	Importance	Performance
PAWR	0.033	70.50
SUV	0.464	71.75
SAVKSI	0.292	81.00

Importance values represent the total effects of the exogenous constructs on study engagement. Because no mediating path was specified in the dimension-level model, total effects correspond to the direct structural paths. Performance values were manually rescaled from the construct means using the theoretical 1–5 response range of the individual interest scale: [(mean−1)/4] × 100.

PAWR, positive affect and willingness to reengage; SAVKSI, stored attainment value and knowledge-seeking intention; SUV, stored utility value.

## Discussion

The present study strengthens the theoretical position that individual interest in PE is not reducible to enjoyment alone, but operates as a multidimensional motivational condition through which students attach affective, practical, and self-relevant meaning to a movement-based activity. In the context of ballroom-based PE, engagement is not only a matter of participation or compliance, but a fuller state of involvement marked by energy, dedication, and absorption in learning. This interpretation aligns with Roure et al. (19) tripartite model, which frames individual interest through positive affect and willingness to reengage, stored utility value, and stored attainment value and knowledge-seeking intention, while also resonating with broader interest-development scholarship that treats interest as both affective and cognitive in nature (19, 20, 43, 44). It also corresponds with the study-engagement tradition, where engagement is understood as a positive, study-related condition involving vigor, dedication, and absorption rather than simple task attendance (28, 30).

The strongest implication of the pattern observed in this study is that students' engagement in ballroom-based PE appears more closely linked to how they recognize the usefulness of ballroom dance than to whether they merely find it enjoyable. This supports the logic of Expectancy-Value Theory, which argues that learners are more likely to invest in tasks they perceive as useful, valuable, or connected to future goals (21). In ballroom-based PE, utility may be experienced through improved posture, coordination, rhythm, cardiovascular movement, body awareness, social interaction, and health-related benefits. This is consistent with broader PE scholarship showing that PE may contribute to physical, social, affective, and cognitive development when learning experiences are meaningfully designed (31). It also aligns with evidence that ballroom and dance-based activities may support physical fitness, balance, gait function, motivation, social cognition, and perceived cognitive, social, emotional, and physical benefits (59–62).

The relevance of stored attainment value and knowledge-seeking intention further suggests that engagement in ballroom-based PE may deepen when students view ballroom dance as personally meaningful, identity-relevant, or intellectually worth understanding. This moves ballroom beyond the narrow image of a formal performance requirement and positions it as an embodied learning experience where students may explore discipline, expressive confidence, cultural knowledge, self-development, and relational awareness. From a self-determination perspective, this interpretation is compatible with the idea that engagement becomes more durable when students experience autonomy, competence, and relatedness in the learning environment (63). It also resonates with evidence that teachers play a crucial role in supporting the quality of student engagement in PE and that motivational teaching behaviors can be organized around need-supportive practices that foster adaptive motivation (22, 34). In dance education specifically, young people's engagement has been associated not only with pleasure but also with effort, curiosity, commitment, and self-development, suggesting that dance becomes educationally powerful when students experience it as meaningful work rather than mere entertainment (35, 64).

The weaker role of PAWR should not be interpreted as evidence that enjoyment is irrelevant in ballroom-based PE. Rather, it suggests that PAWR may not be sufficient to account for study engagement when ballroom dance is experienced through partner interaction, bodily visibility, technical correction, public performance, and culturally coded expectations about movement. In dance-based PE, students' discomfort may emerge from how dance is organized, assessed, and embodied in class, especially when participation involves visibility, social comparison, and uncertainty about bodily competence (6, 8, 9). These concerns may become more pronounced in ballroom because partnered movement can involve proximity, role expectations, and gendered or heteronormative conventions that shape how students experience participation (12–15). A student may enjoy some aspects of ballroom dance, yet still feel uneasy about dancing with a partner, being watched by classmates, performing prescribed movement roles, or receiving correction in a visible setting. This may help explain why PAWR did not show an independent association with study engagement after SUV and SAVKSI were considered.

Another possible explanation is that PAWR may operate indirectly or conditionally rather than as a direct correlate of engagement. Enjoyment may become more engagement-relevant when students also perceive ballroom dance as useful, personally meaningful, or worth learning. It may also depend on contextual conditions such as perceived competence, psychological safety, partner comfort, autonomy support, peer climate, and the instructor's pedagogical style. This interpretation aligns with SDT, which emphasizes the role of autonomy, competence, and relatedness in supporting engagement, and with PE scholarship showing that teacher behavior and motivational climate shape the quality of students' participation (22, 34, 63). In dance education, engagement has also been linked not only to pleasure, but to effort, commitment, curiosity, and self-development, suggesting that enjoyment may need to be supported by value, competence, and meaningful learning conditions before it becomes engagement-relevant (35, 64). These possible mediation or moderation pathways were not tested in the present model, but they offer a useful direction for future research. Longitudinal or mixed-methods designs may clarify whether PAWR becomes more strongly associated with engagement over time, especially as students gain confidence, familiarity, and relational comfort in ballroom-based PE.

Taken together, the findings suggest that engagement in ballroom-based PE was more closely associated with utility-oriented and self-relevant dimensions of individual interest than with affective enjoyment alone. This pattern supports a more value-centered interpretation of ballroom pedagogy, where students are helped to understand the activity not only as a performance requirement, but as a movement-learning experience connected to posture, rhythm, coordination, social interaction, cultural understanding, confidence, and continued participation in PA. Such interpretation is consistent with motivationally supportive teaching in PE and with dance pedagogy scholarship that foregrounds safety, relevance, learner voice, and meaningful participation (9, 22, 34).

At the same time, broader concepts such as epistemic recognition and epistemic justice should be understood as interpretive and future-oriented extensions of the present findings rather than as constructs directly tested in the model. The present study did not measure students' experiences of epistemic recognition, embodied marginalization, or inclusion in ballroom-based PE. However, the non-significant role of PAWR and the stronger associations of SUV and SAVKSI suggest that future research may examine whether students become more engaged when their embodied discomforts, cultural interpretations, and personal meanings are treated as legitimate parts of learning rather than as resistance or lack of interest. Such work may connect the tripartite model of individual interest with emerging PE scholarship on embodied experience, inclusive pedagogy, and epistemic justice (65–67). The cross-sectional and single-institution nature of the present study requires caution, and the observed associations should be understood as context-bound evidence rather than causal proof.

## Conclusion

This study shows that engagement in ballroom-based PE cannot be understood through enjoyment alone. Using the tripartite model of individual interest, the findings suggest that students' engagement was more strongly associated with how they recognized ballroom dance as useful, personally meaningful, and worth learning than with positive affect and willingness to reengage by itself. In a PE context where ballroom may be viewed as formal, outdated, socially exposing, or disconnected from contemporary student preferences, this pattern is important. It suggests that the pedagogical future of ballroom in HE may depend less on making students simply like the activity and more on helping them see why it matters. The study therefore contributes to PE motivation research by showing how the dimensions of individual interest may operate differently in a partnered, performative, and culturally coded movement context. Ballroom-based PE is not only a site for learning steps, rhythm, or performance routines. It is also a movement-learning space where students negotiate value, confidence, proximity, bodily visibility, social interaction, and personal meaning. The findings should still be interpreted cautiously because the study was cross-sectional, conducted in one institution, and based on self-report data. They identify statistical associations, not causal effects. Even so, the results provide a focused empirical basis for rethinking ballroom-based PE as a value-centered and meaning-oriented learning experience. Future longitudinal, mixed-methods, and intervention-based studies can further examine how students' interest develops over time and how pedagogical conditions may support more meaningful engagement in dance-based PE.

### Implications for pedagogy and practice

The findings offer cautious pedagogical implications for ballroom-based PE. Because SUV and SAVKSI showed stronger associations with study engagement than PAWR, instructors may need to foreground the usefulness, personal relevance, and knowledge value of ballroom dance rather than relying only on enjoyment or performance appeal. In practical terms, this means helping students understand how ballroom may contribute to posture, coordination, rhythm, spatial awareness, confidence, social interaction, cultural understanding, and continued movement learning. These implications should not be read as evidence that such strategies will directly increase engagement, but as pedagogical directions that are consistent with the motivational pattern observed in the present study. The weaker role of PAWR also suggests that making ballroom “fun” may not be sufficient when students experience partner work, public visibility, technical correction, role expectations, or possible embarrassment. Therefore, PE instructors may consider participation structures that reduce unnecessary performance pressure and allow students to engage with ballroom in ways that are safe, meaningful, and developmentally appropriate. Examples may include clearer rationales for learning tasks, flexible partner arrangements, progressive skill-building, reflective discussion, and opportunities for students to connect ballroom with their own movement experiences. These practices are offered as interpretive applications of the findings and should be tested more directly in future intervention or classroom-based studies.

### Limitations

Several limitations should be considered in interpreting the findings. First, the study was conducted in a single public university and within one PATH-FIT 4 ballroom dance context. This limits the transferability of the results to other institutions, regions, instructors, and curricular arrangements. The findings should therefore be read as context-bound evidence from a specific HE setting rather than as generalizable evidence for all ballroom-based PE programs. Because ballroom was delivered under a particular institutional and instructional arrangement, students’ responses may have been shaped by the university's PE culture, the assigned instructor's teaching style, class climate, peer dynamics, assessment practices, and curriculum implementation. These contextual features are especially relevant in ballroom-based PE because students' interest and engagement may be shaped not only by the activity itself, but also by how partner work, performance tasks, correction, rhythm, and public visibility are organized in class. Second, although the sample size exceeded the minimum requirement indicated by the *a priori power analysis*, it remains relatively modest for a study that includes HOC assessment, dimension-level structural modeling, and IPMA. Thus, the results should be interpreted as preliminary and context-specific rather than as fully stable estimates for all ballroom-based PE settings. This limitation is especially relevant for the dimension-level model, where the relative associations of the three factors of individual interest with study engagement may vary across larger, more diverse, and multi-institutional samples.

Third, the cross-sectional design prevents causal interpretation. Although the structural models identified statistically significant associations and explained variance, the design does not establish temporal order among the variables. Therefore, the findings should not be interpreted as evidence that individual interest produces or causes study engagement. It is also possible that engaged students become more likely to recognize ballroom dance as useful, personally meaningful, or worth learning. A longitudinal design would be needed to examine how the three factors of individual interest and study engagement change across the duration of a ballroom-based PE course. Fourth, the study did not include a longitudinal perspective. Although individual interest is theoretically understood as a motivational construct that may develop, stabilize, or change through repeated engagement with learning content, the present study measured PAWR, SUV, SAVKSI, and study engagement only after students had completed their formal ballroom dance exposure. This design does not allow examination of how students' affective responses, perceived utility, attainment value, knowledge-seeking orientation, or engagement changed across the semester. Future longitudinal research may track these dimensions at multiple points during the course to determine whether students become more engaged as ballroom becomes more familiar, meaningful, or personally relevant.

Finally, the reliance on self-report measures may have introduced response bias, especially because ballroom dance involves social exposure, body image concerns, partner interaction, and performance-related vulnerability. The study also did not directly examine several possible confounding or contextual factors, including prior dance experience, motor skill level, initial motivation for course participation, gender identity, sexual orientation, cultural beliefs about ballroom, peer climate, and instructional style. These factors may have shaped both students' individual interest and study engagement, making it possible that some of the observed associations were partly influenced by unmeasured learner, classroom, or instructional conditions. Hence, future studies may include these variables as covariates, moderators, or explanatory constructs to clarify how interest and engagement operate in ballroom-based PE.

### Future research directions

Future studies may extend this work by examining how individual interest and study engagement develop across time in ballroom-based PE. Longitudinal designs would be useful in determining whether students' affective responses, perceived utility, attainment value, and knowledge-seeking orientation change as they gain more exposure to ballroom instruction. Mixed-methods studies may also provide richer insight into why some students find ballroom meaningful while others experience discomfort, hesitation, or disengagement. Intervention-based research may examine the effects of inclusive ballroom pedagogies such as role-switching, same-gender pairing options, reflective journals, values-based discussions, trauma-informed facilitation, and culturally responsive lesson framing. Future research may also compare ballroom with other dance genres or movement-based activities to determine whether the same motivational pattern holds across different PE contexts. Finally, integrating the tripartite model of individual interest with constructs such as physical literacy, embodied confidence, psychological safety, epistemic recognition, and inclusive pedagogy may offer a deeper understanding of how students come to experience movement as meaningful educational knowledge.

### Positioning Philippine research in the global discourse

This study contributes to global PE scholarship by situating a motivational model within the cultural and curricular realities of Philippine higher education. Rather than treating the Philippine context as a peripheral site for applying imported theories, the study shows how local PE experiences can generate insights relevant to broader discussions on motivation, engagement, dance pedagogy, and movement-based learning. Ballroom dance, often examined through performance, competition, or Western social dance traditions, is reinterpreted here as a pedagogical space where students negotiate usefulness, identity, cultural meaning, bodily comfort, and engagement. This positioning matters because epistemic recognition in PE also involves acknowledging that students and teachers from non-dominant contexts can contribute to theory-building, not merely provide local examples of already established frameworks. In this sense, Philippine scholarship can help expand how the field understands dance-based PE, especially in relation to culturally responsive teaching, inclusive movement practice, and value-centered engagement. Therefore, the study affirms the capacity of Filipino PE contexts to contribute meaningfully to international conversations on how movement education can become more humane, relevant, and epistemically just.

## Data Availability

The raw data supporting the conclusions of this article will be made available by the authors, without undue reservation.
